# Phase 1 trial of ADI-PEG20 plus cisplatin in patients with pretreated metastatic melanoma or other advanced solid malignancies

**DOI:** 10.1038/s41416-020-01230-8

**Published:** 2021-03-05

**Authors:** Shuyang Yao, Filip Janku, Vivek Subbiah, John Stewart, Sapna Pradyuman Patel, Ahmed Kaseb, Shannon Neville Westin, Aung Naing, Apostolia Maria Tsimberidou, David Hong, Sarina Anne Piha-Paul, Nai Shi, Amanda Johnston, John Bomalaski, Siqing Fu

**Affiliations:** 1 Departments of Investigational Cancer Therapeutics, The University of Texas MD Anderson Cancer Center, Houston, TX USA; 2 Department of Thoracic Surgery, Xuanwu Hospital, Capital Medical University, Beijing, China; 3 Departments of Pathology, The University of Texas MD Anderson Cancer Center, Houston, TX USA; 4 Departments of Melanoma Medical Oncology, The University of Texas MD Anderson Cancer Center, Houston, TX USA; 5 Departments of Gastrointestinal Medical Oncology, The University of Texas MD Anderson Cancer Center, Houston, TX USA; 6 Departments of Gynecologic Oncology and Reproductive Medicine, The University of Texas MD Anderson Cancer Center, Houston, TX USA; 7 Polaris Pharmaceuticals, Inc., San Diego, CA USA

**Keywords:** Cancer, Cancer therapy

## Abstract

**Background:**

Arginine depletion interferes with pyrimidine metabolism and DNA damage-repair pathways, and pairing arginine deiminase pegylated with 20,000-molecular-weight polyethylene glycol (ADI-PEG20) with platinum enhances cytotoxicity in vitro and in vivo in arginine auxotrophs.

**Methods:**

This single-centre, Phase 1 trial was conducted using a 3 + 3 dose escalation designed to assess safety, tolerability and determine the recommended Phase 2 dose (RP2D) of ADI-PEG20.

**Results:**

We enrolled 99 patients with metastatic argininosuccinate synthetase 1 (ASS1) deficient malignancies. We observed no dose-limiting toxic effects or treatment-related mortality. Three percent of patients discontinued treatment because of toxicity. After treatment, 5% (5/99) of patients had partial responses, and 41% had stable disease. The median progression-free and overall survival durations were 3.62 and 8.06 months, respectively. Substantial arginine depletion and citrulline escalation persisted in most patients through weeks 24 and 8, respectively. Tumour responses were associated with anti-ADI-PEG20 antibody levels at weeks 8 and 16 (*p* = 0.031 and *p* = 0.0357, respectively).

**Conclusion:**

Concurrently administered ADI-PEG20 and cisplatin had an acceptable safety profile and had shown antitumour activity against metastatic ASS1-deficient solid tumours. Further evaluation of this treatment combination is warranted.

## Background

Cancer cells may undergo metabolic reprogramming to overcome the extreme energy requirements for their rapid proliferation. The resultant abnormal metabolic pathways in these cancer cells are of great interest as potential therapeutic targets for cancer treatment. Arginine-auxotrophic tumours have abnormal metabolic pathways that cause cancer cells to have a nutritional requirement for arginine.

Arginine is a semi-essential amino acid involved in the regulation of numerous cellular processes like cell signalling, proliferation (by modulating polyamine and nucleotide synthesis), vasodilatation (via nitric oxide) and hormone synthesis.^1–4^ Arginine also plays a crucial role in immune-system regulation.^5–7^ Most normal human cells synthesise arginine from citrulline via two key enzymes, argininosuccinate synthase 1 (ASS1) and argininosuccinate lyase. However, some cancer cells, such as those in melanoma^8^ and hepatocellular carcinoma (HCC),^9^ are deficient in the necessary enzymatic pathways and must instead obtain arginine from the blood to grow and survive. Therefore, in patients with arginine-auxotrophic tumours, depleting arginine from the blood can control tumour growth, induce autophagy followed by cell death via caspase-independent apoptosis, and even eliminate arginine-requiring cancers without damaging normal cells.

The enzyme arginine deiminase (ADI) degrades dietary arginine and enhances cell kills in select tumour cells. ADI pegylated with 20,000-molecular-weight polyethylene glycol (ADI-PEG20) retained highly potent antitumour efficacy against ASS1-negative cancer cells in vivo and in vitro.^10,11^ Early human studies have demonstrated that ADI-PEG20 is safe.^8,12–14^ However, a recent large randomised study comparing treatment with ADI-PEG20 and that with a placebo in patients with sorafenib-refractory advanced HCC demonstrated that treatment with ADI-PEG20 was not associated with improvement in progression-free survival (PFS) or overall survival (OS) durations, although a trend of improved OS for those with more prolonged arginine depletion.^15^ Thus, the clinical use of ADI-PEG20 as a single agent in nonselective patients is hampered by lack of sustained arginine depletion, especially in cancer cells without ASS1 deficiency.

Newer studies with ADI-PEG20 are focused on extending arginine depletion and other potentially synergistic effects in combination with other cancer therapies. Some basic-research studies have tested ADI-PEG20 combined with cisplatin in treatment of malignant melanoma and HCC cells,^16,17^ and ADI-PEG20 plus cisplatin may synergistically kill HCC cells in two ways. First, in ASS1-positive HCC cell lines, cisplatin-based treatment downregulated ASS1,^17^ making the cells sensitive to ADI-PEG20. Second, ADI-PEG20-mediated depletion of nitric oxide inhibited cancer cell proliferation and enhanced cisplatin-induced apoptosis.^18^

No third-line systemic therapies have been proven to extend OS in patients with metastatic melanoma or other advanced ASS1-deficient malignancies. Accordingly, there is a critical need for treatments for patients in whom first- and second-line antitumour treatments have failed. Thus, we conducted this pharmacodynamic Phase 1 trial of ADI-PEG20 and cisplatin in patients with pretreated metastatic melanoma or other advanced ASS1-deficient malignancies and the main aims were to evaluate the safety, tolerability and efficacy of this combination treatment.

## Methods

In this single-centre, open-label, Phase I clinical trial, the safety, tolerability, and efficacy of ADI-PEG20 combined with cisplatin (NCT01665183) were assessed in patients with pretreated metastatic melanoma or other advanced malignancies. The study consisted of two Phases: a dose-escalation Phase, in which a 3 + 3 design was employed to define the dose-limiting toxicity, and recommended Phase 2 dose (RP2D); and a dose-expansion Phase (cohort B = hepatocellular carcinoma (HCC) with biliary tract carcinoma (BTC) or BTC alone; cohort C = cutaneous melanoma; cohort H = HCC; cohort O = ovarian carcinoma; cohort U = uveal melanoma), in which toxicity and efficacy were analysed further. The study was reviewed and approved by the Institutional Review Board at The University of Texas MD Anderson Cancer Center, and all patients signed Institutional Review Board-approved informed consent forms before study enrolment.

### Patient eligibility

Patients 18 years of age or older were eligible for the study if they had a histologically confirmed advanced solid tumour. Additional criteria included an Eastern Cooperative Oncology Group performance status of 0 or 1 and adequate haematological, hepatic and renal function, defined by creatinine clearance of at least 55 mL/min, AST and ALT less than 2.5 × the upper limit of normal, total bilirubin ≤1.2 mg/dl, serum uric acid ≤8 mg/dL, platelets greater than 75,000 cells/mm^3^, and absolute neutrophil count of 1500 cells/mm^3^. Specific criteria for patients with HCC alone or with coexistent BTC included intact hepatic function with a Child-Pugh score of A-B7. Exclusion criteria included systemic anticancer therapy within 4 weeks of study entry, active bleeding within the prior 3 months, uncontrolled or progressing brain or spinal cord metastases, significant concomitant or uncontrolled intercurrent illness, allergy to platinum or to pegylated or *Escherichia coli* products, and previous ADI-PEG20-based therapy.

### Treatment plan

Dose escalation occurred using a standard 3 + 3 design with a 4th dose cohort, followed by a 5th and 6th if tolerated. Each new dose level cohort would be entered 28 days (1 cycle) after the last patients had been entered in the prior cohort. There was no intrapatient dose escalation. Enrolled patients had received the doses as described in Supplementary 1.

A dose-limiting toxicity (DLT) was defined any of the following events that occurred within the initial 28 days of study entry: hematologic events (including grade 4 neutropenia > 7 days duration, febrile neutropenia of any duration with temperature ≥ 38.5 °C, grade 4 anaemia, which requires transfusion, grade 4 thrombocytopenia which requires transfusion) and grade 3 or 4 nonhematologic toxicity that occur during the first cycle that are attributed as possibly, probably or definitely related to the study treatment.

If the next cycle was delayed more than 3 weeks, the patient would be removed from the study. Patients were monitored for DLTs in cycle one. Patients were evaluable if they had received at least three doses of ADI-PEG20 and two doses of cisplatin. Nonevaluable patients (those not receiving at least three doses of ADI-PEG20 and two doses of cisplatin) were to be replaced. Before proceeding to the next dose level, the first three patients in each cohort would have received at least 28 days of treatment.

The dose-escalation scheme had been performed as: (1) If none of the initial three patients in a cohort experience DLT, then a new cohort of three patients would be treated at the next higher dose level. (2) If one of the three patients in a cohort experienced DLT, then up to three additional patients would be treated at the same dose level. Escalation would continue if only one of the six patients experienced DLT. (3) If two or more patients in a cohort experienced DLT, then the maximum tolerated dose (MTD) would have been exceeded, and no further dose escalation would occur. The previous dose level would be considered as the MTD. (4) If only three patients were treated at a dose level under consideration as the MTD, then the MTD Expansion Cohort would be opened. If two or more patients in that cohort experienced DLT, then the previous dose level would be studied in the same fashion.

Safety was assessed according to the Common Terminology Criteria for Adverse Events (version 4.0), and tumour response was assessed according to Response Evaluation Criteria in Solid Tumors 1.1. Therapy continued until toxicity became prohibitive, tumours progressed, or patients withdrew from the study.

### Pharmacodynamics and immunogenicity

In the first 22 enrolled patients, ASS testing on tumour tissue had been performed greater than 14 days prior to receiving first dose of study drug and these patients met the ASS deficient inclusion criterion (defined as 5% ASS expression). Since about 90% of melanoma patients being enrolled would be deficient, ASS deficiency was no longer required under the justification. ASS1 protein expression levels were assessed via immunohistochemistry using an anti-ASS1 antibody at 1:100 dilution as described previously.^19^ Peripheral blood samples were obtained before each treatment (weekly for 8 weeks and then monthly), and patients’ circulating arginine and citrulline levels were determined via liquid chromatography-mass spectrometry. Titres of anti-ADI-PEG20 antibodies were performed once during weeks 1, 4, 5, 6, 7 and 8 and then monthly for patients remaining in the study. Plasma anti-ADI-PEG20 antibody levels were assessed via an enzyme-linked immunosorbent assay.^14^

### Statistical analysis

As described above, this study was performed using a 3 + 3 design, i.e. an additional three patients were allowed per treatment dose level, as needed, for safety assessment. Descriptive summary statistics were used to characterise demographics, safety and antitumour activity. Categorical data were summarised using frequencies and percentages. Continuous data were summarised using median values with 95% confidence intervals (CIs) and ranges. Differences in categorical variables were assessed using the Fisher exact tests. The PFS and OS durations were estimated using the Kaplan–Meier method. The PFS duration was defined as the time from the first day of the study treatment to the date of death or progression (whichever was first). Surviving patients without evidence of progression were censored at the date of their last radiographic assessment of progression. The OS duration was defined as the time from the first day of the study treatment to that of death or April 26, 2017, at which time the patients’ data were censored. Log-rank tests were used to compare PFS- and OS-duration distributions between groups. In assessment of translational correlatives of ADI-PEG20 pharmacodynamics and efficacy outcomes, a nonparameter Mann–Whitney U test was used. Statistical inferences were based on two-sided tests at a significance level of *P* < 0.05. Statistical analyses were carried out using SAS software (version 9.4).

## Results

### Patient demographics

We enrolled 99 patients (23 during dose escalation and 76 during dose expansion) in the study from October 2012 to April 2016 (Table 1). We monitored all patients from the day of study entry to death or April 26, 2017. Prior therapies and procedures related to cancer treatment were common in this pretreated population, and included surgery (100%), chemotherapy (92%), radiotherapy (53%) and concomitant treatment (100%). All 99 patients received at least one dose of study treatment, and the most common reason for discontinuation was disease progression (81%).

**Table 1:** Baseline demographic data.

Patient characteristics	Number of patients (%)
Median age, years (range)	62 (21–82)
Sex	
Female	52 (53)
Male	47 (47)
Race	
Non-Asian	76 (77)
Asian	23 (23)
Tumour history	
Hepatocellular (HCC)	21 (21)
HCC + biliary tract carcinoma	9 (9)
Ovarian carcinoma	16 (16)
Cutaneous melanoma	24 (24)
Uveal Melanoma	13 (13)
Others	16 (16)
Stage	
III or lower	15 (15)
IV	84 (85)
Prior radiology	
Yes	52 (53)
No	47 (47)
Prior chemotherapy or systemic therapy	
Yes	91 (92)
No	8 (8)
Prior cisplatin-based treatment	
Yes	26 (26)
No	73 (74)

### Safety and tolerability

We evaluated all patients to assess treatment safety and tolerability. We observed no dose-limiting toxic effects, the dose level with ADI-PEG20 at 36 mg/m^2^ and for cisplatin at 30 mg/m^2^ was chosen for dose expansion. The final RP2D was established based on safety, tolerance, antitumour activity and optimal biological effects. The most common adverse events (AEs) in this study (Table 2) were myelosuppression, nausea and fatigue, and other common treatment-related AEs included increased creatinine levels and vomiting. Eighteen percent of the patients experienced grade 1/2 events and 76% experienced grade 3/4 events (mainly haematological toxic effects) (Table 3) as their highest level of AEs. We identified no new safety signals in this study.

**Table 2:** AEs reported in more than five patients.

	Dose-escalation cohorts, *N* (%)						RP2D-expansion cohorts, *N* (%)					
	1	2	3	4	5A	5B	H	B	C	O	U	
Patient characteristics	*N* = 3	*N* = 6	*N* = 5	*N* = 3	*N* = 2	*N* = 4	*N* = 22	*N* = 9	*N* = 16	*N* = 20	*N* = 9	All patients, *N* (%)
Anaemia	1 (33)	2 (33)	3 (60)	1 (33)	0	2 (50)	12 (55)	5 (56)	11 (69)	10 (50)	6 (67)	53 (54)
Leukopenia	0	2 (33)	1 (20)	1 (33)	2 (100)	4 (100)	6 (27)	3 (33)	11 (69)	14 (70)	6 (67)	50 (51)
Neutropenia	1 (33)	2 (33)	1 (20)	2 (67)	2 (100)	4 (100)	6 (27)	2 (22)	9 (56)	16 (80)	5 (56)	50 (51)
Thrombocytopenia	1 (33)	2 (33)	5 (100)	2 (67)	2 (100)	3 (75)	7 (32)	3 (33)	11 (69)	8 (40)	6 (67)	50 (51)
Nausea	0	4 (67)	2 (40)	2 (67)	1 (50)	3 (75)	9 (41)	3 (33)	7 (44)	6 (30)	5 (56)	42 (42)
Fatigue	0	3 (50)	3 (60)	1 (33)	1 (50)	3 (75)	9 (41)	1 (11)	6 (38)	6 (30)	6 (67)	39 (39)
Hypoalbumonaemia	0	1 (17)	1 (20)	1 (33)	1 (50)	0	5 (23)	2 (22)	5 (31)	7 (35)	3 (33)	26 (26)
Hyponatremia	1 (33)	1 (17)	2 (40)	1 (33)	2 (100)	0	4 (18)	3 (33)	4 (25)	6 (30)	2 (22)	26 (26)
Constipation	0	2 (33)	0	1 (33)	0	1 (25)	6 (27)	2 (22)	5 (31)	3 (15)	4 (44)	24 (24)
Hyperkalaemia	0	1 (17)	0	1 (33)	0	0	5 (23)	2 (22)	3 (19)	6 (30)	6 (67)	24 (24)
Vomiting	0	4 (67)	3(60)	1 (33)	0	1 (25)	4 (18)	1 (11)	3 (19)	3 (15)	0	20 (20)

*B* HCC with BTC or BTC alone, *C* cutaneous melanoma, *H* HCC, *O* ovarian carcinoma, *U* uveal melanoma.

**Table 3:** Grade 3/4 AEs reported in more than five patients.

	Dose-escalation cohorts, *N* (%)						RP2D-expansion cohorts, *N* (%)					
	1	2	3	4	5A	5B	H	B	C	O	U	
Patient characteristics	*N* = 3	*N* = 6	*N* = 5	*N* = 3	*N* = 2	*N* = 4	*N* = 22	*N* = 9	*N* = 16	*N* = 20	*N* = 9	All patients, *N* (%)
Any grade 3/4 AE (highest severity)	2 (67)	4 (67)	4 (80)	2 (67)	2 (100)	3 (75)	16 (73)	8 (89)	11 (69)	16 (80)	7 (78)	75 (76)
Neutropenia	0	1 (17)	1 (20)	1 (33)	2 (100)	3 (75)	2 (9)	2 (22)	6 (38)	11 (55)	3 (33)	32 (32)
Leukopenia	0	0	1 (20)	0	2 (100)	1 (25)	2 (9)	2 (22)	1 (6)	6 (30)	2 (22)	17 (17)
Anemia	1 (33)	0	1 (20)	0	0	0	5 (23)	2 (22)	3 (19)	2 (10)	0	14 (14)
Thrombocytopenia	1 (33)	1 (17)	2 (40)	0	2 (100)	0	0	2 (22)	1 (6)	2 (10)	0	11 (11)
Hyponatremia	0	0	0	1 (33)	1 (50)	0	1 (5)	1 (11)	2 (13)	2 (10)	1 (11)	9 (9)
Decreased neutrophil count	0	0	0	0	0	0	5 (23)	4 (44)	0	0	0	9 (9)
Fatigue	0	1 (17)	2 (40)	0	0	0	0	0	3 (19)	1 (5)	0	7 (7)
Dyspnea	0	0	1 (20)	0	1 (50)	0	1 (5)	0	0	2 (10)	1 (11)	6 (6)

*B* HCC with BTC or BTC alone, *C* cutaneous melanoma, *H* HCC, *O* ovarian carcinoma, *U* uveal melanoma.

We observed severe AEs in 38 (38%) patients. The only severe AEs considered to be related to the study treatments were grade 3 nausea and vomiting, both of which occurred in one patient in the cohort of patients with HCC and BTC or with BTC alone and were thought to be related to ADI-PEG20 and cisplatin. Three (3%) patients discontinued study treatments due to unacceptable toxicity. Five patients (one with ovarian cancer and four with HCC) died while on the study. These patients experienced fatal AEs thought to be due to tumour progression and unrelated to the treatment.

We considered injection site and hypersensitivity (allergic) reactions to be AEs of special interest, as ADI is a non-human protein and ADI-PEG20 is administered by the intramuscular route. Overall, injection site-related events were uncommon and included erythema (2%), pain (2%), injection site reactions (2%) and discomfort (1%). No patients discontinued treatment due to these events. No cases of anaphylaxis were observed.

### Efficacy analysis

Table 4 summarises the best overall responses by cohort and for the entire patient population. For all patients, the best overall responses were partial responses in 5% of patients, stable disease in 41% and progressive disease in 28%. The remaining patients did no undergo post-baseline tumour assessments; most discontinued study treatment prior to the imaging assessment in week 8 because of clinical disease progression. Of the five patients who experienced partial responses, two had cutaneous melanoma, one had uveal melanoma, one had ovarian carcinoma and one had HCC. The overall response rate was 5%.

**Table 4:** Best overall responses of all cohorts.

	Dose-escalation cohorts, *N* (%)						Dose-expansion cohorts, *N* (%)					
	1	2	3	4	5A	5B	H	B	C	O	U	
Best overall responses	*N* = 3	*N* = 6	*N* = 5	*N* = 3	*N* = 2	*N* = 4	*N* = 22	*N* = 9	*N* = 16	*N* = 20	*N* = 9	All subjects, *N* (%)
Partial response	0	0	0	1 (33)	0	1 (25)	1 (5)	0	1 (6)	1 (5)	0	5 (5)
Stable disease	1 (33)	3 (50)	2 (40)	0	0	3 (75)	8 (36)	6 (67)	5 (31)	7 (35)	6 (67)	41 (41)
Progressive disease	1 (33)	1 (17)	1 (20)	1 (33)	0	0	7 (32)	1 (11)	8 (50)	7 (35)	1 (11)	28 (28)
Not evaluable	1 (33)	2 (33)	2 (40)	1 (33)	2 (100)	0	6 (27)	2 (22)	2 (13)	5 (25)	2 (22)	25 (25)

*B* HCC with BTC or BTC alone, *C* cutaneous melanoma, *H* HCC, *O* ovarian carcinoma, *U* uveal melanoma.

**Fig. 1:**
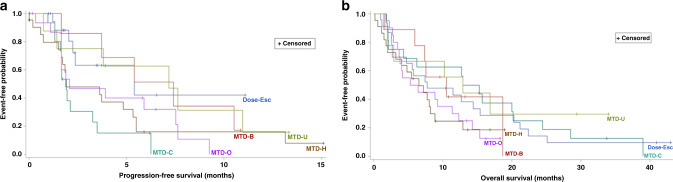
Survival curves according to the ADI-PEG20 with cisplatin in all cohorts. **a** Progression-free survival curve; **b** Overall survival curve. Dose-Esc dose-escalation cohorts (combined for this analysis), MTD-B HCC-with-BTC or BTC-alone cohort, MTD C cutaneous melanoma cohort, MTD-H HCC cohort, MTD-O ovarian carcinoma cohort, MTD-U uveal melanoma cohort.

Of the 99 patients, 82% experienced disease progression or died as of the last date of study contact. The median PFS duration was 3.62 months (95% CI, 1.94–5.39 months) (Fig. 1a). Seven patients had durable responses/disease control for >10.0 months (range, 10.5–15.1 months). The median OS duration was 8.06 months (95% CI, 5.95–12.30 months) (Fig. 1b). Of note, the longest PFS and OS durations occurred in the uveal melanoma cohort.

### Pharmacodynamics and immunogenicity analyses

**Fig. 2:**
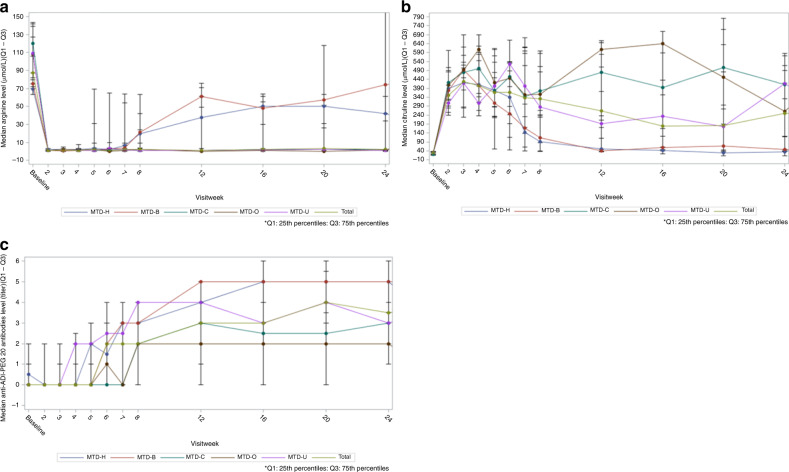
Pharmacodynamic results of ADI-PEG20 with cisplatin in all cohorts. **a** Median arginine levels in blood by timepoint; **b** Median citrulline levels in blood by timepoint; **c** Anti-ADI-PEG20 antibody levels in blood by timepoint. MTD-B HCC-with-BTC or BTC-alone cohort, MTD C cutaneous melanoma cohort, MTD-H HCC cohort, MTD-O ovarian carcinoma cohort, MTD-U uveal melanoma cohort, Q1 quartile 1, Q3 quartile 3.

Median levels of arginine declined rapidly after the first weekly dose of study treatment (87.0 µM baseline to 1.0 µM Week 2 for the total population), indicative of ADI-PEG20 activity. Overall, 98% of subjects experienced arginine depletion (defined as a negative change from baseline and ≤10 µM) by Week 2. This response was durable, with arginine levels remaining below baseline through Week 7. By Week 8, some recovery of arginine levels was seen for the cohort H and cohort B, both of which showed variable, yet increasing levels of arginine through Week 24 (Fig. 2a). As expected, based on these arginine findings, the median citrulline levels in the entire study population increased rapidly after the first treatment dose (28.7 µM at baseline to 351.0 µM predose week 2). Citrulline levels remained elevated through week 8 (Fig. 2b), after which results were more variable. The median anti-ADI-PEG20 antibody levels began to rise as early as week 3 in the uveal melanoma cohort, with all MTD cohorts having a titre of at least 2 by week 8. Median anti-ADI-PEG20 antibodies remained above baseline through week 24 (Fig. 2c). The antibody levels were undetectable in the responders, but present in the nonresponders. Using a nonparameter Mann–Whitney *U* test to compare responders with nonresponders within the entire patient population, we found an association between tumour responses and anti-ADI-PEG20 antibody levels at weeks 8 and 16 (*p* = 0.031 and *p* = 0.0357, respectively). However, the presence of the anti-ADI-PEG20 antibody had a minimal effect on arginine and citrulline levels. In the dose-expansion cohorts, we found no association between tumour response and PFS or OS duration when we examined patients’ arginine and citrulline levels.

## Discussion

In this Phase 1 study, ADI-PEG20 plus cisplatin had an acceptable safety profile and encouraging antitumour activity in patients with selected solid tumours. The RP2D was 36 mg/m^2^ ADI-PEG20 weekly for 4 weeks with 30 mg/m^2^ cisplatin given weekly for 3 weeks of every 4-week cycle. The safety profile for ADI-PEG20 plus cisplatin was consistent with that observed in prior ADI-PEG20-related trials, in that we observed no unexpected AEs. We did note, however, that the proportion of grade ≥3 toxic effects in our study was much higher than that of other studies with ADI-PEG20 monotherapy.^8,11,14^ The primary reason for this is most likely due to cisplatin, which is well known to be associated with toxic events, including bone marrow suppression. In addition, it may be due to our patients having undergone heavy treatment before their enrolment in our study so that they had worse organ functions and suffered more AEs from the combination treatment. It is also unclear whether this result represents a true biologic phenomenon or a consequence of a limited research numbers. Nevertheless, only 3% patients discontinued study treatment due to an AE. In general, toxicities were manageable with supportive care medications, treatment interruption and discontinuation, and/or dose reductions.

In regard to the antitumour activity of the treatment, we observed that 5% of patients had partial responses and 41% had stable disease. Some other promising study results demonstrated that the addition of cytotoxic therapy to ADI-PEG20 led to an modest doubling of the response rate in ASS1-deficient malignant tumours.^20–22^ The enrolled patients in those studies were primarily chemotherapy naïve and thus more likely to benefit from the cancer treatment. Taken together, the data support the hypothesis that arginine depletion might potentiate the cytotoxic effects of certain chemotherapeutics in both ASS1-proficient and deficient cancers. Although we did observe responses in patients with recurrent platinum resistant malignancies, the relatively small number of objective responses observed in this study might be simply attributed to cisplatin.

Our pharmacodynamic data demonstrated that arginine was suppressed at the end of the first cycle of treatment for all patients and that ADI-PEG20 depleted plasma arginine levels throughout the 24-week treatment period to levels below 10% of baseline in the majority of patients (Fig. 2a). As expected, median citrulline levels increased rapidly after the first treatment dose and remained elevated through week 8, after which the levels were variable. These results were not consistent with those of prior studies in which patients with advanced solid tumours underwent ADI-PEG20-based monotherapy and had normal arginine and citrulline concentrations 8 and 12 weeks after first dosage.^12,14^

We observed a continuous increase in anti-ADI-PEG20 antibody induction over time, as seen in other combination chemotherapy studies with ADI-PEG20 in patients with mesothelioma, non-small-cell lung cancer, HCC, and other gastrointestinal malignancies.^20,21,23^ Like previous researchers, we did not observe any association of the presence of the anti-ADI-PEG20 antibody with blood levels of arginine or citrulline. However, we observed an association between tumour response and anti-ADI-PEG20 antibody levels at weeks 8 and 16, suggesting that the presence of this antibody might be a potential predictive marker for ADI-PEG20 and cisplatin antitumour efficacy, which needs further confirmation.

In considering the clinical relevance of our findings, some limitations of our study should be kept in mind. The selection bias associated with our eligibility criteria may limit the generalisability of our findings, as is the case for many clinical trials. Also, the small sample sizes in our subgroup analyses limited the validity of the statistical assessments of individual pathological diagnoses.

In conclusion, our findings support the theory that ASS1-deficient cancer cells have many biological advantages to promote cell survival and mediate resistance to chemotherapy that may be reversed by arginine depletion.^11,24,25^ Our preliminary clinical findings demonstrate that further evaluation of treatment with ADI-PEG20 plus cisplatin and the use of an anti-ADI-PEG20 antibody as a potential marker for antitumour efficacy is warranted in patients with metastatic melanoma and many other ASS1-deficient malignancies.

## Supplementary information

Supplementary Table S1

## Data Availability

All data supporting the results in this manuscript are available at Polaris Pharmaceuticals, Inc.
