# Supplementary material for: Phase 1 trial of ADI-PEG20 plus cisplatin in patients with pretreated metastatic melanoma or other advanced solid malignancies
Source: Br J Cancer. 2021 Mar 5;124(9):1533–9. doi: 10.1038/s41416-020-01230-8 (PMC8076217; doi:10.1038/s41416-020-01230-8)
Supplement: Supplementary file 1 — Supplementary Table S1 [file 41416_2020_1230_MOESM1_ESM.docx]

Supplementary Table S1: Dose-escalation scheme

| **Cohort** | ***N*** | **Cisplatin dose (mg/m^2^) QW Weeks 1 - 3** | **ADI-PEG 20 dose (mg/m^2^) QW  Weeks 1 - 4** |
| --- | --- | --- | --- |
| 1 | 3-6 | 20 | 18 |
| 2 | 3-6 | 25 | 18 |
| 3 | 3-6 | 30 | 18 |
| 4 | 3-6 | 35 | 18 |
| 5A | 3-6 | 30 | 36 |
| 5B | 3-6 | 35 | 36 |

Abbreviation: QW = once weekly.

Supplementary table 1: Dose-escalation scheme.
